# The Presence of Hemoglobin in Cervicovaginal Lavage Is Not Associated With Genital Schistosomiasis in Zambian Women From the BILHIV Study

**DOI:** 10.1093/ofid/ofac586

**Published:** 2022-12-16

**Authors:** Amy S Sturt, Emily L Webb, Comfort R Phiri, Joyce Mapani, Maina Mudenda, Lisa Himschoot, Eyrun F Kjetland, Tobias Mweene, Bruno Levecke, Govert J van Dam, Paul L A M Corstjens, Helen Ayles, Richard J Hayes, Suzanna C Francis, Lisette van Lieshout, Piet Cools, Isaiah Hansingo, Amaya L Bustinduy

**Affiliations:** Department of Infectious Diseases, Veterans Affairs Health Care System, Palo Alto, California, USA; MRC International Statistics and Epidemiology Group, London School of Hygiene and Tropical Medicine, London, United Kingdom; Zambart, Lusaka, Zambia; Department of Obstetrics and Gynecology, Livingstone Central Hospital, Livingstone, Zambia; Department of Obstetrics and Gynecology, Livingstone Central Hospital, Livingstone, Zambia; Department of Diagnostic Sciences, Faculty of Medicine and Health Sciences, Ghent University, Ghent, Belgium; Department of Infectious Diseases and Global Health, Norwegian Centre for Imported and Tropical Diseases, Oslo University Hospital, Oslo, Norway; Discipline of Public Health Medicine, College of Health Sciences, University of Kwa-Zulu Natal, Durban, South Africa; Zambart, Lusaka, Zambia; Department of Translational Physiology, Infectiology and Public Health, Ghent University, Merelbeke, Belgium; Department of Parasitology, Leiden University Medical Center, Leiden, The Netherlands; Department of Cell and Chemical Biology, Leiden University Medical Center, Leiden, The Netherlands; Zambart, Lusaka, Zambia; Department of Clinical Research, London School of Hygiene and Tropical Medicine, London, United Kingdom; MRC International Statistics and Epidemiology Group, London School of Hygiene and Tropical Medicine, London, United Kingdom; MRC International Statistics and Epidemiology Group, London School of Hygiene and Tropical Medicine, London, United Kingdom; Department of Parasitology, Leiden University Medical Center, Leiden, The Netherlands; Department of Diagnostic Sciences, Faculty of Medicine and Health Sciences, Ghent University, Ghent, Belgium; Department of Translational Physiology, Infectiology and Public Health, Ghent University, Merelbeke, Belgium; Department of Obstetrics and Gynecology, Livingstone Central Hospital, Livingstone, Zambia; Department of Clinical Research, London School of Hygiene and Tropical Medicine, London, United Kingdom

**Keywords:** cervicovaginal lavage, female genital schistosomiasis, hematuria, hemoglobin, *Schistosoma haematobium*

## Abstract

**Background:**

Female genital schistosomiasis (FGS) occurs when *Schistosoma haematobium* eggs are deposited in reproductive tissue. Female genital schistosomiasis in the cervical mucosa is associated with increased vascularity. If FGS is associated with the presence of hemoglobin in cervicovaginal lavage (CVL), the use of urinary reagent strips to detect hemoglobin in CVL could supplement FGS diagnosis.

**Methods:**

Nonmenstruating, nonpregnant, sexually active women aged 18–31 participating in the HPTN 071 (PopART) Population-Cohort were invited in 2 Zambian communities. Genital self-swabs and a urine specimen were collected at a home visit, and CVL and hand-held colposcopy were performed at a midwife led clinic visit. Urinary reagent strips were used to identify hemoglobin in CVL. Eggs and circulating anodic antigen (CAA) were detected from urine. Visual-FGS was defined as the presence of sandy patches, rubbery papules, or abnormal blood vessels. Polymerase chain reaction (PCR)-FGS was defined as *Schistosoma* deoxyribonucleic acid detected by real-time PCR on CVL or cervical or vaginal swab.

**Results:**

Of 209 women with home genital swabs and companion CVL specimens, 66% (138 of 209) had detectable CVL hemoglobin, 13.4% (28 of 209) had PCR-defined FGS, and 17.2% (36 of 209) had visual-FGS. Active *Schistosoma* infection, diagnosed by CAA or urine microscopy, was present in 21.0% (44 of 209) participants. Active *Schistosoma* infection (*P* = .4), PCR-FGS (*P* = 0.7), and visual-FGS (*P* = 0.3) were not associated with CVL hemoglobin presence. Results did not differ in subgroups with high infection burden (cycle threshold < 35 or 2–3 positive genital PCR).

**Conclusions:**

Polymerase chain reaction-FGS, visual-FGS, and active *Schistosoma* infection were not associated with the presence of CVL hemoglobin. Further research is needed to establish accessible community-based FGS diagnostics.

## Teaser

Urinary reagent strips (URS) are useful for estimating *S haematobium* prevalence. Female genital schistosomiasis (FGS) occurs as a complication of *S haematobium* infection and is associated with cervical mucosal changes. This study evaluates URS in cervicovaginal lavage for FGS diagnosis.

It is estimated that over 80 million African women are living with *Schistosoma haematobium* infection [1]. Female genital schistosomiasis (FGS) is caused when *S haematobium* eggs destined for excretion in the urinary bladder are deposited in reproductive tissue [2]. Female genital schistosomiasis of the uterine cervix has been associated with characteristic clinical manifestations, such as sandy patches (homogenous and grainy), rubbery papules, and abnormal blood vessels [3]. The presence of parasite eggs in reproductive tissues may be associated with inflammation, fibrosis, and infertility [4, 5]. Parasite egg deposition in reproductive tissue may also lead to mucosal friability and vascularization. For example, the presence of *S haematobium* eggs in the pap smears of Zimbabwean women has been associated with contact bleeding [3]. In addition, the presence of *S haematobium* in the cervices of Malawian women has been associated with a higher density of blood vessels than in the cervices of Norwegian controls [6].

Establishing a diagnosis of FGS is challenging due to the lack of an appropriate reference standard, and currently both molecular and visual techniques are used. Molecular methods that detect *S haematobium* deoxyribonucleic acid (DNA), such as polymerase chain reaction (PCR) and recombinase polymerase assay, are not yet widely available in resource-limited settings [7, 8]. Although molecular methods have imperfect sensitivity, they show promise as highly specific FGS diagnostics [7, 9]. Although visual diagnosis, using portable colposcopy, is scalable and potentially more accessible than molecular methods, it suffers from a lack of specificity. Characteristic cervicovaginal sandy patches identified on portable colposcopy can also be associated with sexually transmitted infections (STIs), and abnormal blood vessels have been associated with cervical intraepithelial neoplasia 1–3 [3]. Urine microscopy and urine circulating anodic antigen (CAA) are useful diagnostic tools for diagnosing active *Schistosoma* infection, but they cannot confirm genital involvement. Urinary reagent strips are affordable, user-friendly, rapid, equipment free, and widely available diagnostics used in estimating the community prevalence of urinary schistosomiasis: hematuria is often used as a surrogate marker of *S haematobium* infection [10–12]. If FGS is associated with the presence of hemoglobin in cervicovaginal lavage (CVL) fluid, the use of urinary reagent strips to detect hemoglobin in CVL could supplement existing FGS diagnostic methods. However, the association of *Schistosoma* infection and FGS with CVL hemoglobin has not previously been described. The overall aim of this study is to evaluate whether the presence or absence of hemoglobin in CVL is associated with FGS and *Schistosoma* infection status.

## METHODS

### Study Setting and Participants

The cross-sectional bilharzia and HIV (BILHIV) study [9] was nested in HPTN 071 (PopART), a cluster-randomized trial to measure the impact of an HIV-1 combination prevention package [13]. As previously described, between January and August 2018, after the 36-month HPTN 071 (PopART) visit, community workers made home visits to women expressing interest in the BILHIV study [9]. Eligible women were 18–31 years old, not pregnant, sexually active, and resident in 1 of 2 urban communities that participated in HPTN 071 (PopART) in Livingstone, Zambia.

### Home- and Clinic-Based Sample and Image Collection

The home visit included written informed consent, a questionnaire, genital self-sampling (cervical and vaginal), and urine specimen collection, as previously described [9]. Questionnaires were administered by a field worker. Self-collected vaginal and cervical swab specimens were used for *Schistosoma* quantitative polymerase chain reaction (qPCR), and self-collected cervical swabs were used for characterization of STI by qPCR (Table 1). Urine specimens were used for detection of CAA and *S haematobium* eggs by microscopy (Table 1). Enrolled women who were not currently menstruating were then invited to attend Livingstone Central Hospital cervical cancer clinic, where midwives collected CVL. After speculum insertion, a bulb syringe was used to flush normal saline (10 mL) across the cervix and vaginal walls for 1 minute. Cervicovaginal lavage fluid was collected from the posterior fornices.

**Table 1.** Specimens Collected and Procedures Performed in the BILHIV Study

Specimen or Procedure	Diagnostic Test	Home Visit	Clinic Visit
Urine	…	X	…
	Circulating Anodic Antigen	…	…
	Urine microscopy	…	…
	Urine reagent strip	…	…
Vaginal Swab	…	X	…
	*Schistosoma* PCR	…	…
	…	…	…
Cervical Swab	…	X	…
	*Schistosoma* PCR	…	…
	STI diagnostics	…	…
Cervicovaginal Lavage	…	…	X
	*Schistosoma* PCR	…	…
	Urine reagent strip	…	…
Portable Colposcopy	…	…	X
	Cervicovaginal image capture	…	…

Abbreviations: BILHIV, bilharzia and HIV study; PCR, polymerase chain reaction.

At the clinic, cervicovaginal images were captured with a portable colposcope (MobileODT, Tel Aviv, Israel) and were evaluated by an expert reviewer (E.F.K.) for any of the 4 recognized FGS cervicovaginal manifestations: grainy sandy patches, homogenous yellow sandy patches, rubbery papules, and abnormal blood vessels [14]. Women with at least 1 of the visual manifestations of FGS [3, 14] or with any positive urine or genital *Schistosoma* diagnostic were treated free-of-charge with 40 mg/kg praziquantel. In line with national and local clinic protocols adapted to real-world resource limitations, human papillomavirus (HPV) testing was not performed. Cervical self-sampling for STIs was performed at the home visit and STI diagnostics were not done at the point-of-care. As per local guidelines, participants with suspected STIs were offered syndromic management [15].

Separate from BILHIV study procedures, participants could choose to engage in free cervical cancer screening using the visual inspection with acetic acid (VIA) technique. In the subset of women who engaged in screening, midwives applied 3%–5% acetic acid to the cervix after CVL collection, as previously described [16]. An opaque white reaction was classified as positive and no change was negative [17].

### Cervicovaginal Lavage Fluid Processing

Cervicovaginal lavage fluid was stored at −80°C for a maximum of 20 months (range, 12–20), as previously described [18, 19]. Previously unthawed CVL specimens were centrifuged at 320 *×g* for 10 minutes and the supernatant was removed. A 10-μL aliquot of CVL supernatant was placed on a Multistix test strip (Siemens, Munich, Germany). As per the manufacturer's instructions, CVL hemoglobin concentrations were recorded after comparing the test strip with color categories representing approximate quantities of erythrocytes (ery) per microliter: none, trace, small (25 ery/μL); moderate (80 ery/μL); large (200 ery/μL) [18]. Cervicovaginal lavage fluid was considered positive for hemoglobin when any hemoglobin (at a level of trace or above) was detected in CVL fluid.

### Urine Microscopy, Reagent Strip Testing, and Circulating Anodic Antigen

Up to 50 mL fresh urine was centrifuged and examined by microscopy for *S haematobium* eggs. The participant was considered to have urinary schistosomiasis if a pellet contained at least 1 *S haematobium* egg [9]. Hematuria was detected using Multistix test strip according to the manufacturer's instructions and was defined as the detection of any hemoglobin (at a level of trace or above) in urine. A lateral flow assay utilizing up-converting reporter particles for the quantification of CAA was performed on urine samples, as previously described [9, 20]. Analyzing the equivalent of 417-μL urine (wet reagent, UCAA***hT***417), a test result indicating a CAA value >0.6 pg/mL was considered positive [21].

### Quantitative Polymerase Chain Reaction for Detection of *Schistosoma* Deoxyribonucleic Acid

Detection of the *Schistosoma*-specific internal-transcribed-spacer-2 (ITS-2) target by qPCR was performed at Leiden University Medical Center, as previously described [9, 22]. Deoxyribonucleic acid extraction of 200 µL CVL, cervical, or vaginal swab fluid was done with QIAamp spin columns (QIAGEN Benelux, Venlo, The Netherlands) according to manufacturer's guidelines. The qPCR output was reported in cycle threshold (Ct) values, and parasite DNA loads were categorized by the following prespecified values: high (Ct < 30), moderate (30 ≤ Ct < 35), low (35 ≤ Ct < 50), and negative (no amplification) [23].

### Other Clinical Characteristics

Laboratory-based, fourth-generation HIV-1 testing (Abbott Architect HIV Ag/Ab Combo Assay) was performed for HPTN 071 (PopART) Population Cohort participants at each study visit [13].

Sexually transmitted infections were quantified by qPCR using the S-DiaCTNG (for *Chlamydia trachomatis* and *Neisseria gonorrhea*) and S-DiaMGTV kits (for *Mycoplasma genitalium* and *Trichomonas vaginalis*) (Diagenode Diagnostics, Seraing, Belgium) on DNA from cervical swabs at Ghent University (Ghent, Belgium) according to the manufacturer's instructions.

### Female Genital Schistosomiasis and Schistosomiasis Definitions

In this study, we used various diagnostic tests to evaluate urinary *Schistosoma* infection (CAA and urine microscopy) and FGS (portable colposcopy, and *Schistosoma* DNA on CVL and genital swabs) as previously described [19, 24, 25]. As well as analyzing each diagnostic method separately for association with the outcome of this analysis (presence of CVL hemoglobin), we also used case definitions to characterize participants. Thus, participants were also grouped by diagnostic test results into 3 mutually exclusive categories: (1) FGS, at least 1 positive *Schistosoma* qPCR on a genital specimen (cervical swab, vaginal swab, and/or CVL); (2) “negative” FGS, negative results on all diagnostic methods; (3) “probable” FGS, negative genital *Schistosoma* qPCR but urinary schistosomiasis positive (as defined above), in combination with at least 1 of 4 clinical findings suggestive of FGS on any colposcope-obtained photograph [14]. Active *Schistosoma* infection was defined as detection of either CAA or *S haematobium* eggs in urine.

### Statistical Methods

As previously described, all participants with FGS (*n* = 28) and all participants with probable FGS (*n* = 24) were selected for hemoglobin testing in CVL. Three FGS negative participants were selected for every FGS and probable FGS participant using a random number generator. The FGS negative participants were frequency matched by 7 age bands (18–19, 20–21, 22–23, 24–25, 26–27, 28–29, 30–31) to participants with FGS, resulting in a 3:1 ratio of FGS negative to FGS and probable FGS.

Participant characteristics were summarized by median and interquartile range for continuous variables and by frequency and percentage for categorical variables. Differences in baseline characteristics by CVL hemoglobin status were evaluated using Fisher's exact, χ^2^, or Kruskal-Wallis tests.

The primary analysis evaluated the association of FGS (PCR-defined or visually defined) and urinary schistosomiasis with the presence versus absence of CVL hemoglobin. Logistic regression was used to calculate crude and adjusted odds ratio for presence versus absence of CVL hemoglobin with the relevant FGS or *Schistosoma* infection definition. We adjusted for age, education, and community of residence and did not adjust for STIs because this variable is not consistently associated with FGS [24]. Data were analyzed using STATA 15.1 (Stata Corporation, College Station, TX).

### Patient Consent Statement

The study was approved by the University of Zambia Biomedical Research Ethics Committee (011-08-17), the Zambia National Health Research Authority, and the London School of Hygiene and Tropical Medicine Ethics Committee (14506). Permission to conduct the study was given by Livingstone District Health Office and the Livingstone Central Hospital superintendent. Participants provided written informed consent.

## RESULTS

**Figure 1.**
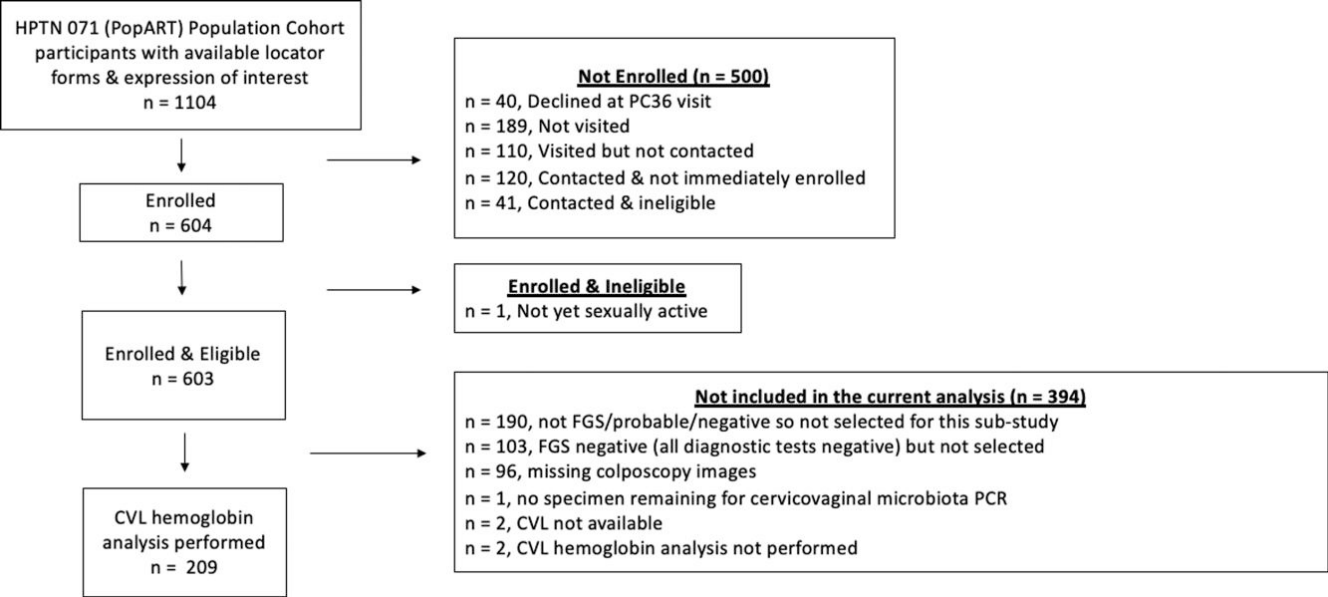
Flowsheet of the study participants (*n* = 209). CAA, circulating anodic antigen; CVL, cervicovaginal lavage; FGS, female genital schistosomiasis; HPTN, HIV Prevention Trial Network; PC, population cohort; PCR, polymerase chain reaction; STI, sexually transmitted infection.

The BILHIV study enrolled 603 eligible women, 209 (34.6%) of whom were included in the current analysis (Figure 1). Polymerase chain reaction-FGS was diagnosed in 13.4% (28 of 209) of participants, defined by a positive genital *Schistosoma* qPCR from any of the following sites: 8.6% (18 of 209) cervical swab, 6.7% (14 of 209) vaginal swab, and 6.7% (14 of 209) CVL. Visual-FGS was detected in 17.3% (36 of 208) of participants by expert review of digital images from hand-held colposcopy with 11.5% (24 of 209) having probable FGS. Of the participants who were negative on all diagnostic tests, 59.9% (157 of 262) were randomly selected for inclusion in this study. Of participants with PCR-FGS, 53.3% (15 of 28) had *Schistosoma* qPCR detected from more than 1 specimen (whether swab [cervical or vaginal]) or cervicovaginal lavage, and 53.3% (15 of 28) had moderate/high genital *Schistosoma* DNA loads, as defined by cycle threshold <35. These subgroups with high-burden PCR-FGS overlapped by 11 participants.

The majority of patients (66.0%, 138 of 209) had detectable hemoglobin in their CVL fluid (trace, 15.3% [32 of 209]; small, 19.1% [40 of 209]; moderate, 15.3% [32 of 209]; large, 16.3% [34 of 209]). Compared to participants without hemoglobin detected in CVL, there was no difference in baseline characteristics such as age, marital status, education, or district of residence in participants with hemoglobin present in CVL (Table 2). There was strong evidence that a higher proportion of women with STIs had hemoglobin detected in CVL compared with women without STIs (*P* = 0.04). However, other reproductive health characteristics, such as lifetime number of sexual partners, HIV-1 status, VIA status, and contraceptive use, did not differ by CVL hemoglobin status. One third (34.9%) of participants had at least 1 STI (Table 2). At the conclusion of HPTN 071 (PopART), HIV-1 prevalence in this study population was 17.2% (36 of 209).

**Table 2.** Characteristics of the Study Population (*n* = 209) and Crude Associations With CVL Hemoglobin Status

Participant Characteristics	…	CVL Hemoglobin Negative (*n*)% (*n* = 71)	CVL Hemoglobin Positive (*n*)% (*n* = 138)	*P* Value^a^
	…	…	…	…
Age in years, median (IQR)	…	23 (22–26)	23 (21–25)	.9^b^
Marital Status	Single	31 (35.2)	57 (64.8)	.3^c^
	Married or cohabitating	38 (33.0)	77 (67.0)	…
	Divorced or separated	2 (33.3)	4 (66.7)	…
Education (Highest Level)	None or any primary school	20 (37.0)	34 (63.0)	.8^c^
	Any secondary school	48 (33.6)	95 (66.4)	…
	Trade, degree or higher	3 (25.0)	9 (75.0)	…
District	Community A	32 (30.5)	73 (69.5)	.3
	Community B	39 (37.5)	65 (62.5)	…
Household Members	1–3	22 (33.9)	43 (66.2)	.7
	4–5	30 (37.0)	51 (63.0)	…
	6+	19 (30.2)	44 (69.8)	…
Employment Status	Not working	50 (32.7)	103 (67.3)	.5
	Working	21 (37.5)	35 (62.5)	…
Reproductive Health Characteristics		…	…	…
Age at Sexual Debut (Years)	8–16	36 (40.0)	54 (60.0)	.2
	17–19	29 (30.2)	67 (69.8)	…
	20–24	6 (26.1)	17 (73.9)	…
Lifetime Sexual Partners	1	21 (30.0)	49 (70.0)	.4
	2	14 (28.0)	36 (72.0)	…
	3	14 (40.0)	21 (60.0)	…
	4+	22 (40.7)	32 (59.3)	…
Prior Pregnancy^d^	No	14 (46.7)	16 (53.3)	.2
	Yes	57 (32.0)	121 (68.0)	…
Currently Sexually Active^d,e^	No	11 (35.5)	20 (64.5)	.9
	Yes	60 (33.9)	117 (66.1)	…
Condom Use With Last Sex^f^	No	49 (31.6)	106 (68.4)	.2
	Yes	21 (41.2)	30 (58.8)	…
HIV-1 STATUs	Not detected	55 (31.8)	118 (68.2)	.2
	Detected	16 (44.4)	20 (55.6)	…
Any STIs	Not detected	53 (39.0)	83 (61.0)	.04
	Detected	18 (24.7)	55 (75.3)	…
VIA Status^g^	Acetowhite changes not detected	25 (28.4)	63 (71.6)	.2
	Acetowhite changes detected	4 (50.0)	4 (50.0)	…
Contraceptive Use	…	…	…	…
Condoms	No	57 (32.6)	118 (67.4)	.3
	Yes	14 (41.2)	20 (58.8)	…
Oral contraceptive pill	No	68 (34.9)	127 (65.1)	.4^c^
	Yes	3 (21.4)	11 (78.6)	…
Injectable	No	41 (38.0)	67 (62.0)	.2
	Yes	30 (29.7)	71 (70.3)	…
Implant	No	63 (33.0)	128 (67.0)	.3
	Yes	8 (44.4)	10 (55.6)	…
Any hormonal contraception^h^	No	30 (39.5)	46 (60.5)	.2
	Yes	41 (30.8)	92 (69.2)	…

Abbreviations: CVL, cervicovaginal lavage; HIV, human immunodeficiency virus; IQR, interquartile range; STI, sexually transmitted infection; VIA, visual inspection with acetic acid.

a χ^2^*P* value unless otherwise indicated.

b Kruskal-Wallis *P* value (mean age is presented as a column value, all other variables indicate row percentages).

c Fisher's exact *P* value.

d Participants who responded with “no answer” (*n* = 1) are not shown in the table.

e Any sexual activity in the last 6 months.

f Participants who responded with “no answer” (*n* = 3) are not shown in the table.

g VIA results were available for *n* = 96 participants.

h Any hormonal contraception is defined as use of injectable agents, implants, or oral contraceptive pills.

*Schistosoma haematobium* eggs were detected in urine microscopy in 12.0% (25 of 209) and CAA was detectable in 20.1% (42 of 209), and 21.1% (44 of 209) of participants had either positive urine microscopy or detectable CAA. Hematuria, detected by urine reagent strip, was present in 34.5% (72 of 209) of participants. There was no association between hematuria and the presence of hemoglobin in CVL (*P* = .9). All participants with eggs detected on urine microscopy (12.0%, 25 of 209) had hematuria (*P* < .001), whereas 76.2% (32 of 42) of participants with a detectable CAA had hematuria by urine reagent strip (*P* < .001).

**Figure 2.**
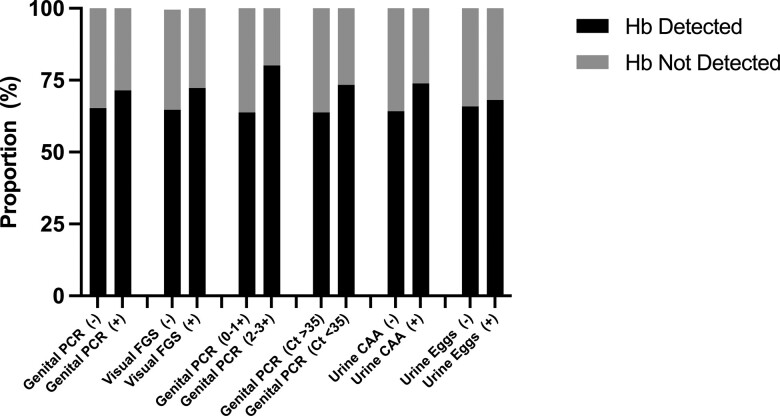
Stacked bar chart of presence or absence of hemoglobin (Hb) in cervicovaginal lavage fluid by FGS or *S haematobium* infection status. CAA, circulating anodic antigen; Ct, cycle threshold; FGS, female genital schistosomiasis; PCR, polymerase chain reaction.

The crude and adjusted association of presence and absence of CVL hemoglobin was evaluated by FGS status (Table 3). There was no evidence that either PCR-based or visually based methods (eg, portable colposcopy) of FGS detection were associated with the presence of CVL hemoglobin. There was no evidence that site and method of PCR-based FGS detection (whether cervical or vaginal, swab, or CVL) was associated with the presence of CVL hemoglobin (Figure 2). In addition, predefined FGS categories (definite or probable FGS) were not associated with the presence of CVL hemoglobin. A higher burden of FGS, whether defined by 2–3 positive genital PCR specimens, or lower PCR cycle threshold (Ct < 35), was not associated with the presence of hemoglobin in CVL. The crude and adjusted association of presence and absence of CVL hemoglobin was evaluated by urinary schistosomiasis status. There was no evidence that active *S haematobium* infection, as diagnosed by either positive urine microscopy for *S haematobium* eggs or detectable CAA, was associated with the presence of hemoglobin in CVL.

**Table 3.** Crude and Adjusted Associations of the Presence and Absence of CVL Hemoglobin by FGS and *Schistosoma* Infection Status in the Study Population (*n* = 209), Adjusted for Age, Education, and District of Residence

Participant Characteristics	…	CVL Hemoglobin Negative (*n*)% (*n* = 71)	CVL Hemoglobin Positive (*n*)% (*n* = 138)	Crude OR	*P* Value^a^	Adjusted OR	*P* Value
FGS and *Schistosoma* Status	…	…	…	…	…	…	…
Cervical Swab PCR	DNA not detected	65 (34.0)	126 (66.0)	Reference	1.0	Reference	.9
	DNA detected	6 (33.3)	12 (66.7)	1.03 (0.37–2.87)	…	0.95 (0.33–2.70)	…
Vaginal swab PCR	DNA not detected	68 (34.9)	127 (65.1)	Reference	.4^b^	Reference	.3
	DNA detected	3 (21.4)	11 (78.6)	1.96 (0.53–7.28)	…	1.92 (0.51–7.19)	…
Cervicovaginal lavage PCR	DNA not detected	69 (35.4)	126 (64.6)	Reference	.2^b^	Reference	.1
	DNA detected	2 (14.3)	12 (85.7)	3.29 (0.71–15.10)	…	3.11 (0.67–14.52)	…
Visual FGS^c^	Lesion not detected	60 (34.9)	112 (65.1)	Reference	.3	Reference	.4
	Lesion detected	10 (27.8)	26 (72.2)	1.39 (0.63–3.08)	…	1.44 (0.61–3.43)	…
FGS category	Negative FGS	57 (36.3)	100 (63.7)	Reference	.5	Reference	.4
	Probable FGS	6 (25.0)	18 (75.0)	1.71 (0.64–4.55)	…	1.96 (0.68–5.63)	…
	FGS	8 (28.6)	20 (71.4)	1.43 (0.59–3.44)	…	1.35 (0.54–3.36)	…
PCR FGS (2-3 positive specimens)	0–1 PCR detected	57 (36.3)	100 (63.7)	Reference	.2	Reference	.2
	2–3 PCR detected	3 (20.0)	12 (80.0)	2.28 (0.62–8.42)	…	2.31 (0.62–8.64)	…
PCR FGS (Ct <35)	PCR Ct >35	57 (36.3)	100 (63.7)	Reference	.5	Reference	.4
	PCR Ct <35	4 (26.7)	11 (73.3)	1.57 (0.48–5.15)	…	1.61 (0.48–5.42)	…
Urine microscopy	Not detected	63 (34.2)	121 (65.8)	Reference	.5	Reference	.9
	Detected	8 (32.0)	17 (68.0)	1.11 (0.45–2.70)	…	1.04 (0.41–2.60)	…
Urine CAA	Not detected	60 (35.9)	107 (64.1)	Reference	.2	Reference	.2
	Detected	11 (26.2)	31 (73.8)	1.58 (0.74–3.37)	…	1.64 (0.73–3.67)	…

Abbreviations: CAA, circulating anodic antigen; Ct, cycle threshold; CVL, cervicovaginal lavage; DNA, deoxyribonucleic acid; FGS, female genital schistosomiasis; IQR, interquartile range; OR, odds ratio; PCR, polymerase chain reaction; STI, sexually transmitted infection.

a χ^2^*P* value unless otherwise indicated.

b Fisher's exact *P* value.

c Participants (*n* = 1) with images that could not be evaluated are not shown in the table.

## DISCUSSION

Urinary reagent strips are rapid, user-friendly diagnostics used to detect hematuria, an indirect marker of *S haematobium*-related morbidity [11, 26, 27]. Because cervical tissue in women with genital schistosomiasis may be more vascularized than that of women without genital schistosomiasis [6], we evaluated whether identifying hemoglobin in CVL fluid might be associated with either FGS or *Schistosoma* infection status. Regardless of the FGS definition (whether PCR-FGS or visual-FGS) or the method of *Schistosoma* infection diagnosis (whether by CAA or urine microscopy), we found that neither FGS status nor *Schistosoma* infection were associated with CVL hemoglobin detection.

As described by King et al [11], in *S haematobium*-endemic areas, urine reagent strips are frequently used as a predictor of egg-patent *S haematobium* infection, or to provide an estimate of probable *S haematobium* infection. Compared with urine microscopy, urine reagent strips require less equipment and are easier to perform, while still being relatively inexpensive [11, 28]. However, in the community diagnosis of schistosomiasis, the use of urinary reagent strips is challenging in women of reproductive age because hemoglobin can be detected in urine due to other reasons, such as pregnancy, menstruation, and sexually transmitted infection [26]. For example, a Tanzanian study estimated *S haematobium*-related hematuria in women of reproductive age (aged 15–50) compared with urine microscopy as a reference standard. In 3 study areas with *S haematobium* prevalence of 3%, 5%, and 53% the authors of the Tanzanian study report thathematuria, detected by urinary reagent strip, was present in 18%–38% of women not excreting *S haematobium* eggs [26]. In addition, only 54% of hematuria could be attributed to *S haematobium*, and 10%–17% of women without *S haematobium* egg excretion had hematuria detected in the intermenstrual period [26]. Although urine microscopy is an imperfect reference standard and has limited sensitivity in low-prevalence areas [29], the Tanzanian study nevertheless illustrates challenges with specificity when using urinary reagent strips in women of reproductive age [26]. Furthermore, the presence of hemoglobin in CVL may be common in women of reproductive age. For example, a Tanzanian cohort of reproductive-age women found that 67% of participants had some degree of CVL hemoglobin detection [18]. The BILHIV study participants self-reported their pregnancy and menstruation status, so it is possible that undetected pregnancy or residual menstrual blood may have influenced the presence of hemoglobin in CVL. Because STIs cause cervicitis, it is not surprising that CVL hemoglobin was detected in a higher proportion of women with STIs in this study compared to women without STIs. Because FGS has not been routinely associated with STIs, adjustment for STIs is not presented in this analysis. However, inclusion of STIs in the model did not significantly change the presented findings (data not shown). The presence and absence of STIs will need to be considered if the detection of CVL hemoglobin is to be taken forward in future work with FGS. In addition, because there are limited data on the performance of reagent strip testing for detecting hemoglobin on fresh versus frozen CVL, in future work, researchers may wish to consider performing reagent strip testing on CVL at the point-of-care.

Participant age, sex, infection intensity [30], and *S haematobium* prevalence [29] may affect urine reagent strip test performance. Before sample selection for this study, the overall prevalence of *Schistosoma* infection in the BILHIV study was 5.5% by urine microscopy, thus meeting World Health Organization classification of a low-risk community [31]. Because ours is the first study evaluating the association between the presence of CVL hemoglobin and FGS, it is unclear how *S haematobium* and FGS burden may affect reagent strip test performance when used in CVL. Further research will be needed to investigate the association between CVL hemoglobin and FGS in a setting with a higher *S haematobium* prevalence and FGS burden.

Using urine reagent strips to detect CVL hemoglobin in the setting of FGS has not been previously described. Although this approach is novel, this study has some limitations. Although CVL is often performed in research settings, there are limitations to the routine use of CVL for FGS diagnosis. Cervicovaginal lavage is a relatively invasive procedure requiring trained medical staff, a suitable clinical setting, and vaginal speculum insertion. The BILHIV study was cross-sectional, limiting the evaluation of multiple time-points or the long-term impact of different FGS definitions [9]. In addition, the *S haematobium* prevalence was relatively low, and thus the numbers of FGS cases were small, limiting precision in the effect sizes. Thus, we cannot conclusively exclude an association between FGS and the detection of CVL hemoglobin. The BILHIV study participants self-reported their pregnancy and menstruation status, so it is possible that undetected pregnancy or residual menstrual blood may have influenced the presence of hemoglobin in CVL. In addition, because cervical cancer and/or precancerous lesions are among the causes of cervical bleeding [32], it is a limitation that we did not have information on HPV infection prevalence in this cohort. Visual inspection with acetic acid data were not obtained within the BILHIV study, thus data on this variable are incomplete [16]. Given these factors, we cannot exclude residual or unmeasured confounding. We did not evaluate genital specimens for prostate-specific antigen to evaluate the presence of recent sexual contact. Thus, we cannot exclude that cervicovaginal qPCR detected *S haematobium* DNA in eggs present in the semen of a sexual partner [9]. Because the BILHIV study participants self-collected genital swabs, we cannot exclude sampling error nor falsely negative genital swabs. In future studies using self-collected specimens, β-globin PCR could be used as a positive control to confirm the presence of human DNA [9].

## CONCLUSIONS

In conclusion, although urinary reagent strips are a useful diagnostic tool in evaluating the community prevalence of *S haematobium* infection, in our study PCR-FGS, visual-FGS, and active *Schistosoma* infection were not associated with the presence of CVL hemoglobin. This manuscript identifies a knowledge gap regarding the performance of urinary reagent strips as a supplement to CVL in FGS diagnosis, specifically in study populations with a higher FGS burden. However, given the limitations of performing CVL in routine FGS diagnosis, alternate diagnostic strategies are needed. Isothermal DNA amplification methods are field-deployable molecular assays that could be used at the point-of-care to detect *Schistosoma* DNA in self-collected genital swabs [7]. Further research and support are required to make this and other community-based FGS diagnostics available and accessible.
