# Correction to: The Presence of Hemoglobin in Cervicovaginal Lavage Is Not Associated With Genital Schistosomiasis in Zambian Women From the BILHIV Study

**DOI:** 10.1093/ofid/ofae472

**Published:** 2024-08-22

This is a correction to: Amy S Sturt, Emily L Webb, Comfort R Phiri, Joyce Mapani, Maina Mudenda, Lisa Himschoot, Eyrun F Kjetland, Tobias Mweene, Bruno Levecke, Govert J van Dam, Paul L A M Corstjens, Helen Ayles, Richard J Hayes, Suzanna C Francis, Lisette van Lieshout, Piet Cools, Isaiah Hansingo, Amaya L Bustinduy, The Presence of Hemoglobin in Cervicovaginal Lavage Is Not Associated With Genital Schistosomiasis in Zambian Women From the BILHIV Study, *Open Forum Infectious Diseases*, Volume 9, Issue 12, December 2022, ofac586, https://doi.org/10.1093/ofid/ofac586

In the originally published version of this manuscript, an error was made and Amaya L Bustinduy was omitted as co-corresponding author. This error has been corrected to reflect Amaya L Bustinduy as co-corresponding author alongside, Amy S Sturt.

